# Characteristics of traumatic out-of-hospital cardiac arrest patients presenting to major centers in Karachi, Pakistan—a longitudinal cohort study

**DOI:** 10.1186/s12245-018-0214-7

**Published:** 2018-11-22

**Authors:** Minaz Mawani, Masood Kadir, Iqbal Azam, Junaid Abdul Razzak

**Affiliations:** 1 Department of Medicine, The Aga Khan University, First floor Faculty Offices Building, Stadium Road, P.O. Box 3500, Karachi, 74800 Pakistan; 2 Department of Community Health Sciences, Aga Khan University, Karachi, Pakistan; 3 Global Emergency Medicine, Johns Hopkins University School of Medicine, Baltimore, USA

**Keywords:** Traumatic out-of-hospital cardiac arrest, Pre-hospital care, Survival

## Abstract

**Background:**

Trauma is the leading cause of death for adults under 44 years of age. Survival after traumatic out-of-hospital cardiac arrest (OHCA) has been reported to be poor, and its epidemiology is not well defined. A few studies have reported better survival in response to pre-hospital life-saving interventions. Currently, no published data on traumatic cardiac arrests in the field exist from low- and lower middle-income countries. We aimed to explore the epidemiology and outcomes of traumatic OHCA patients from Karachi, Pakistan.

We conducted a longitudinal cohort study at emergency departments (ED) of five major public and private hospitals of the city from January to April 2013. Data was collected on all adult patients (age 18 years or more) presenting to the hospitals directly from field with cardiac arrest and history of trauma using a structured questionnaire. Patients with do-not-resuscitate status and those referred from other hospitals were excluded.

**Results:**

During 3 months, a total of 187 patients were enrolled with mean age of 35.1 years. About 95% were men, and 68.4% had a penetrating injury. Even though half of the patients had a witnessed arrest, none received a bystander cardiopulmonary resuscitation (CPR). 83.4% were brought to the hospital in an ambulance, with median response and scene times of 3 and 2 min respectively; however, only 3 received any pre-hospital life-support interventions. One hundred eighty-one patients (96.7%) were pronounced dead on arrival to the ED, and of the remaining 6 patients, 4 received CPR in the EDs. Overall survival at the end of ED stay was 0%. Patients who received life-support interventions survived for longer time, though not clinically significant, as compared to those who did not (45 min vs. 35 min, *p* = 0.02).

**Conclusion:**

There was no survival after a traumatic OHCA in Karachi, Pakistan. Even though ambulances reached the scene in a very short time, pre-hospital interventions were largely absent. There is a strong need to strengthen our pre-hospital care system but most importantly train the general public to deal with emergencies and be able to provide timely bystander CPR.

## Background

Trauma is the leading cause of death for adults in their productive years, i.e., under 44 years of age, and responsible for more years of life lost compared to chronic diseases like stroke, cardiovascular diseases, and cancer combined [1]. In low- and middle-income countries such as Pakistan, injuries are among the top 10 causes of mortality especially among young individuals and cause about 42 deaths per 100,000 population [2]. Lack of pre-hospital and hospital-based trauma care system is often mentioned as a major contributor to the poor injury outcomes in low-resource settings [3].

Standard pre-hospital trauma care comprises of the first responder care that includes activating emergency medical service system and first aid by firefighters, police, and trained lay responders; basic pre-hospital trauma care involving control of external hemorrhage (direct or indirect pressure), protection of spine, provision of artificial respiration, circulatory support, oxygen therapy and extrication, stabilization, and transportation to trauma care facility without causing further harm; and advanced pre-hospital trauma care that includes intravenous fluid therapy, endotracheal intubation, and other invasive interventions such as needle decompression or cricothyroidotomy. A balance between stay and play vs. scoop and run is recommended in trauma patients depending on the distance to trauma center, pre-hospital resources available, and mechanism of injury [4]. The hospital care for trauma, according to the ATLS (advance trauma life support) guidelines, includes airway stabilization, maintaining adequate oxygenation, controlling hemorrhage, and ensuring end organ perfusion; disability assessment; and assessment for possible injuries [5].

Survival with meaningful neurological recovery is poor after traumatic out-of-hospital cardiac arrest (OHCA) [6] though some recent studies have reported survival to hospital discharge of up to 5–7%. The improvement in survival is attributed to pre-hospital life-saving interventions [7–9]. Earlier studies have focused on particular subgroups of injuries and mortality such as road traffic injuries and those resulting from bomb blasts, burns, and falls [10, 11]. However, pre-hospital traumatic cardiac arrest in low- and lower middle-income countries has been relatively unexplored [2]. The purpose of this study is to describe the epidemiology of pre-hospital traumatic cardiac arrest, including its survival rates, and explore the pre-hospital and emergency department care provided to these patients.

## Study methodology

### Study design and setting

This study is part of a larger longitudinal cohort study conducted in five major hospitals in Karachi, Pakistan [12]. Karachi, the largest city in Pakistan, has an estimated population of 20 million [13]. The city has a large number of government and privately funded hospitals although majority of trauma patients still go to three major government trauma centers [14]. The hospitals selected for this study were the largest teaching hospitals of Karachi, catering population from all towns of the city and thereby helped obtain a representative study sample [15]. Four hospitals were public sector, out of which three hospitals were major trauma centers of the city, whereas one was a private not-for-profit teaching hospital. None of these hospitals had a functioning trauma registry.

Pre-hospital care systems in Karachi, Pakistan, are very different than those established internationally. For instance, in developed countries, a single network of emergency medical services (EMS) provides uniform pre-hospital services across the nation with a common emergency contact number such as 911. There are several different ambulance services in Karachi, Pakistan, with varying levels of emergency care services being provided. Only one philanthropic organization has ambulances equipped with trained personnel and equipment for dealing with emergencies whereas others are just providing rapid transportation. Some of the patients in critical condition are even brought to the hospitals in a public or own transportation, whatever is readily available. Due to these differences, the type of pre-hospital transportation for this study was categorized into three categories: “ambulance with life-support interventions” (ambulances with a trained professional to provide pre-hospital care and facilities such as cardiopulmonary resuscitation (CPR), life-saving medications, and automated external defibrillator), “ambulances without life-support interventions” (ambulances destined to provide early transfer to a tertiary care center without any definitive pre-hospital care with equipment consisting of only oxygen cylinder and a stretcher), and “non-EMS transportation” (any private or public transportation, other than ambulance, used to transfer patient to a hospital, i.e., taxi and private car).

### Selection criteria

We included all patients with age 18 years or older presenting to the study sites with a history of traumatic out-of-hospital cardiac arrest. The operational definition of traumatic OHCA was “patients who had an event of unresponsiveness and absence of breathing outside the hospital setting secondary to trauma.” Pulse check was removed from the definition of cardiac arrest based on the fact that pulse check by a lay person is mostly uncertain in correctly diagnosing cardiac arrest and a bystander might refrain from providing CPR when the patient actually requires it. The diagnosis of traumatic OHCA was confirmed by a doctor either in the ambulance or on arrival to the emergency department (wherever a doctor became first available) since not all ambulances had a medical professional in our setting. Excluded were all patients with a do-not-resuscitate status and those who were referred from other hospitals. We also excluded non-traumatic causes such as drowning, electrocution, hanging, poisoning, or any other medical causes. Data was collected from January to April 2013.

### Study questionnaire

The questionnaire was completed by data collectors placed in the emergency departments of the study sites. It comprised of questions pertaining to demographics, arrest-related characteristics, and pre-hospital and hospital interventions. To ensure accurate recording of time intervals for this study, data collectors matched time on their watch with the ones being used at the hospitals and EMS and any discrepancies were incorporated in the analyses accordingly.

The questionnaire was developed in English, translated into Urdu, and translated back into English, and no changes in meanings were observed during the process. These translations were carried out by individuals having command over both the languages and subject as well. Pretesting of the questionnaire was done on 10% of the study sample, and the feasibility of study was checked through a small-scale pilot project conducted in three of the study sites: one private and two public sector hospitals. Two of the data collectors from the team were trained and assigned in 12-h shifts for 3 days in each hospital. The questionnaire was modified based on the results from pilot and pretesting. Some of the modifications were addition of patient’s date of birth; options for location of arrest; type of witness; comorbid conditions; CPR personnel; type of life-support medications given; time of stopping CPR; reasons for not doing or discontinuing CPR; types of pre-hospital transport, i.e., ambulance without life support, ambulance with life support, and non-EMS; questions to ensure clock synchronization (time match between EMS, dispatch center, and data collector’s watch); questions to obtain details on facilities present in each ambulance (trained personnel to provide CPR, defibrillator, life-saving medications, etc.); questions to inquire patient’s status on arrival at ED; questions on advance airway, and other advanced management; and some changes to simplify language of the questionnaire to match laymen proficiency.

### Data collection

A team of 17 data collectors were trained to collect data from three different sources: EMS personnel (if applicable), hospital personnel, and family members/bystanders. The data collectors worked in three 8-h shifts to provide 24-h coverage in the emergency departments of the selected study sites. The study coordinator supervised the project to ensure coverage of all study sites and quality of data collection. Paper-based forms were collected once in 24 h and checked by the study coordinator for completion. Any discrepancies in the data were rechecked from hospital records.

We looked at survival on presentation and discharge from ED, and it was defined as patients with pulse and spontaneous breathing (if not pharmacologically paralyzed) at the time of arrival to and transfer from the emergency department.

### Statistical analysis

Continuous variables with normal distribution such as age were reported as mean and standard deviation. Time to reach hospital, time to interventions, and time to event were reported as median with interquartile range (IQR). Frequencies with percentages were reported for all categorical variables such as gender, cause of arrest, and type of trauma. The rate of survival on arrival and on discharge from ED was calculated from the number of patients surviving at that point divided by the total number of OHCA patients included in the study. Categorical variables were compared across the categories of type of trauma, types of pre-hospital transportation, time to reach hospital, and median survival times using chi-square test. Continuous variables were examined across categories using independent sample *t* test, Mann Whitney *U* test, ANOVA (analysis of variance), or Kruskhal-Wallis test. Mean survival times were compared across the categories using log rank test with Kaplan-Meier survival curves. Results with a *p* value of < 0.05 were considered to be statistically significant. All analyses were carried out using SPSS (Statistical Package for Social Scientists version 19; IBM Corporation, NYC, USA).

### Ethical considerations

The study protocol was reviewed and approved by the Ethics Review Committee of the Aga Khan University and Karachi Medical and Dental College. In addition, permissions were obtained from institutional and departmental heads at each study site. Informed consent was obtained from family members of the participants after explaining the study details.

## Results

### Demographic characteristics of the participants

During the study period, a total of 187 traumatic out-of-hospital cardiac arrest patients presented to the study sites with mean age ± SD (standard deviation) of 35.1 years (11.1). Most of the patients were young, and about 60.4% of the patients belonged to the 18–35-year age category. Most of the victims were men (95.2%), and majority (68.4%) had a penetrating injury, usually a gunshot wound, followed by the road traffic crashes (24.2%). About 8% of the arrest occurred because of a fall, crush injuries, blunt injury due to violence, bomb blasts, and burns (Table 1).

**Table 1:** Characteristics of patients with traumatic OHCA presenting to five major hospitals of Karachi

Characteristics	*n* (%)
Age in years, mean (SD), range 18–70 years	35.1 (11.1)
18–35	113 (60.4)
36–54	60 (32.1)
55–70	14 (7.5)
Gender	
Men	178 (95.2)
Women	9 (4.8)
Type of trauma	
Blunt	58 (31.0)
Penetrating	128 (68.4)
Cause of arrest	
Gunshot	127 (68.3)
RTA	45 (24.2)
Fall	5 (2.7)
Crushed	1 (0.5)
Violence with a blunt object	6 (3.2)
Bomb blast victim	1 (0.5)
Burn	1 (0.5)
Unknown cause	1 (0.5)
Site of arrest	
Residence	14 (7.5)
Public	173 (92.5)
Type of witness	
Lay person	59 (31.6)
Health care provider	1 (0.5)
Family	42 (22.5)
EMS	2 (1.1)
None	83 (44.6)
Type of pre-hospital transportation	
Ambulance with life support	6 (3.2)
Ambulance without life support	150 (80.2)
Non-EMS	31 (16.6)
First assessed rhythm	
Shockable	0 (0)
Non-shockable	140 (74.9)
Not recorded	47 (25.1)
Time to reach hospital, median (IQR)	30.0 (20,40)
Outcomes on arrival at ED	
Alive	6 (3.2)
Declared dead	181 (96.8)
Median survival time (IQR)	35.0 (23.0, 51.0)
Destination hospital	
Public	185 (98.9)
Private	2 (1.1)
Life-saving interventions	
Pre-hospital	3 (1.6)
Hospital	7 (3.7)
None	177 (94.7)
CPR	
Pre-hospital	2 (1.1)
Hospital	6 (3.2)
None	179 (95.7)
Time to the first life-support intervention, median (IQR), range 7–58 min *n* = 10	30 (13.2, 33.5)
EMS scene time, median (IQR), range 0–8 min *n* = 156	2 (1,3)
EMS response time, median (IQR), range 0–20 min *n* = 156	3 (1,6.7)

*SD* standard deviation, *RTA* road traffic accident, *OHCA* out-of-hospital cardiac arrest, *EMS* emergency medical services, *IQR* interquartile range, *CPR* cardiopulmonary resuscitation, *ED* emergency department

### Pre-hospital transportation and interventions

Most of the traumatic arrests occurred on the road/streets 128 (68.4%) followed by workplace 27 (14.4%) and at home 14 (7.5%). Thirteen patients suffered traumatic OHCA during transportation (ambulance 9 (4.8%) and other transportation 4 (2.1%)). Almost half (*n* = 104, 55.6%) of the patients had a witnessed arrest, yet none of the patients received a bystander CPR (cardiopulmonary resuscitation) or a dispatch-assisted CPR. One hundred fifty (80.2%) of the patients were transferred to the hospital in an ambulance without equipment and or trained staff. Only 6 (3.2%) were transported via ambulances with capacity to provide life-support interventions, and 31 (16.6%) were transported via non-EMS (public or private transportation). Upon comparing the characteristics based on the type of transportation, most of the witnessed arrests were transported via non-EMS transportation (83.9% vs. 49.3% for ambulance without life support and 66.7% for ambulance with life-support interventions). The highest percentage of life-support interventions and CPR could be seen among patients utilizing ambulance with life-support interventions as compared to the other two modes of transportation (ambulance with life support = 50%, ambulance without life support 2.7%, and 9.7% for non-EMS, *p* < 0.001). Three patients being transported by the ambulance with life-support interventions received any life-support interventions in the pre-hospital setting, two of them received CPR for 2 and 4 min and one of them received a life-support medication as well (epinephrine); another patient was given atropine but no compressions were initiated. When inquired about the reasons for not performing CPR in the pre-hospital settings, in 148 patients, the reason was that there was no one in the vehicle besides the driver; in 8 cases, death was declared in the EMS, and in one case, it was assessed that there was no need of CPR.

### Response and scene times

Median (IQR) response time for ambulance was 3 min (1, 6.7) whereas median (IQR) scene time was 2 min (1, 3). EMS response time was longer for ambulance with life-support interventions as compared to ambulance without life support (10 min vs. 3 min); however, the difference was not statistically significant. EMS scene times were not different across the categories of ambulances. Median (IQR) time to receive the first life-support intervention was 30 min (13.2, 33.5), and median (IQR) time to reach hospital was 30 min (20, 40). Median time to reach the hospital was shorter for patients arriving by non-EMS transportation (27 min) compared to those arriving by life-support ambulances (32.5 min) and non-life-support ambulances (30 min) (*p* = 0.05) while time to the first life-support intervention was the shortest for ambulance with life-support interventions (14 min vs. 30 min for non-EMS and 36 min for ambulance without life-support interventions, *p* = 0.02). Time to reach hospital was the shortest for patients with RTA (road traffic accident) as compared to gunshot, fall, and others (26 min vs. 30 and 35 min). EMS scene time was not different across the categories; however, EMS response time was the longest for fall and others (5 min vs. 3 min for all other categories, *p* = 0.08).

### Interventions provided in the ED department

96.8% (*n* = 181) patients were declared dead on arrival to the EDs. Six patients received CPR (i.e., chest compressions and life-saving medications) in the hospital setting, 3 of these were transported via ambulance without life-support facilities and 3 were transported via non-EMS. CPR interval in these patients ranged from 3 to 49 min. One of the patients was given life-support medication only (atropine) and was intubated. Overall, four patients were intubated in the hospital ED, using an oral endotracheal tube. None of the participants received any other care. In all 6 cases, CPR was stopped in the ED and death was declared by the treating emergency physician. One hundred thirty (69.5%) had asystole on arrival to the ED, 7 (3.7%) had pulseless electrical activity, and 3 (1.6%) had unknown non-shockable rhythm, and in 47 (25.1%) patients, rhythm was not recorded in the EDs. All of the patients that received life-support interventions in the hospital setting had a witnessed arrest.

### Survival outcomes

**Fig. 1:**
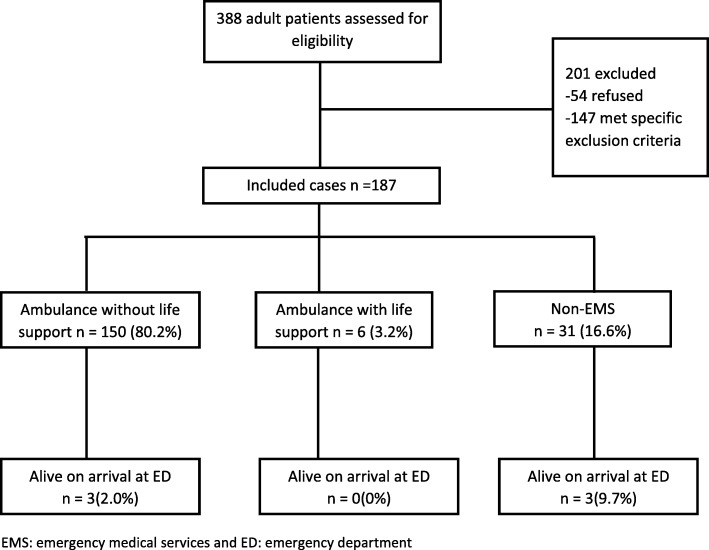
Flow diagram of a multicenter longitudinal cohort study. Outcomes of patients by mode of pre-hospital transportation presenting with out-of-hospital traumatic cardiac arrests to the emergency department of five major hospitals of the city

Although 6 patients did survive on arrival to the ED, none survived to the end of emergency department stay (Fig. 1). Median (IQR) survival time was 35.5 min (23, 51). Compared to blunt injury, median (IQR) survival time was longer for penetrating trauma (44.0 min (38.6–49.5) vs. 38.4 min (30.0–46.8), *p* value = 0.06). In addition, patients who had longer survival times were the ones who received higher percentage of life-support interventions as seen in Table 2. Survival time was the longest for patients with gunshot injury as compared to RTA, fall, and others (40 min vs. 31 and 32 min). Among women, blunt injury was much more common than the penetrating injuries (12.5% vs. 1.6%) whereas survival time was longer for penetrating trauma patients (40 min vs. 32 min). Survival time was not significantly different across the three modes of transportation (41 min for ambulance with life support, 36 min for ambulance without life support, and 35 min for non-EMS).

**Table 2:** Comparison of characteristics based on median survival time (35 min)

Variables	Less than or equal to median survival time *n* = 94	More than median survival time *n* = 93	*p* values
Age in years, mean (SD)	34.5 (10.3)	35.6 (11.9)	0.47
Gender			1.0
Men	89 (94.7)	89 (95.7)	
Women	5 (5.3)	4 (4.3)	
Type of arrest			0.02
Penetrating	57 (62.0)	71 (77.2)	
Blunt	35 (38.0)	21 (22.8)	
Cause of arrest			0.03
Gunshot	56 (60.2)	71 (76.3)	
RTA	30 (32.3)	15 (16.1)	
Fall and others	8 (8.5)	7 (7.5)	
Site of arrest			0.56
Residence	6 (6.4)	8 (8.6)	
Public	88 (93.6)	85 (91.4)	
Life-support interventions			0.01
Yes	1 (1.1)	9 (9.7)	
No	93 (98.9)	84 (90.3)	
Life-support medications			0.01
Yes	1 (1.1)	8 (8.6)	
No	93 (98.9)	85 (91.4)	
CPR			0.03
Yes	1(1.1)	7(7.5)	
No	93(98.9)	86(92.5)	
Outcomes on arrival at ED			0.11
Alive	1 (1.1)	5 (5.4)	
Dead	93 (98.9)	88 (94.6)	
Type of transportation			0.62
Ambulance without life support	75 (79.8)	75 (80.6)	
Ambulance with life support	2 (2.1)	4 (4.3)	
Non-EMS	17 (18.1)	14 (15.1)	

*SD* standard deviation, *RTA* road traffic accident, *EMS* emergency medical services, *CPR* cardiopulmonary resuscitation, *ED* emergency department

## Discussion

This is one of the first studies from low- to middle-income country to describe the out-of-hospital traumatic arrest. Most of the traumatic OHCA victims were young men between 18 and 35 years of age, and none of them survived irrespective of level of pre-hospital life-support interventions. This is consistent with the universal poor survival of post traumatic OHCA. However, some of the studies have reported better survival in response to pre-hospital life-support interventions [16, 17].

We found large gaps in pre-hospital care of the victims. Even though about half of the participants had a witnessed arrest, none of them received a bystander CPR. A study from Australia reported similar findings where traumatic arrests were more likely to occur in a public setting and witnessed however that these were less likely to receive bystander CPR [8]. In our setting, there are several potential reasons for it. Few people are trained in CPR, and even fewer feel comfortable with doing CPR even if trained. The discomfort is potentially exacerbated in patients with gunshot wounds and potential blood on the torso. Also, there is no “good-Samaritan” law in Pakistan which deter people in getting named in a potential “medico-legal case.” Finally, the scene of violence injury is a high-risk site for further violence, and hence, patients are moved to safer locations inside the hospital instead of being treated in the field.

This also reflects unstable city conditions and highlights the need for legislative actions such as licensing of arms and ammunitions and better implementation of traffic policies. A very small number of participants received CPR (two in the pre-hospital setting and six in the hospital setting). Median time to receive the first life-support interventions was 30 min post event. However, patients being brought to the hospital through an ambulance with life-support intervention had a shortest median time to receive life-support intervention (14 min) and also the highest proportion of life-support interventions received, which shows that an ambulance with trained personnel and proper equipment, though not being utilized much, has a potential for saving more lives [18].

We observed very short median response and scene time for ambulances. Karachi has several different EMS networks consisting of a large number of ambulances spread across the city. Since pre-hospital response and scene time are major performance indicators for ambulances, therefore, these ambulances are very quick in responding. However, most of the ambulances only have oxygen cylinder and a stretcher and no trained person to handle medical emergencies. Only 3.2% of the patients in our study were transported to the hospital in an ambulance with trained personnel and life-saving equipment. In the current scenario with such short response times, one can benefit from training ambulance personnel on basic life-support intervention.

## Limitations

Our study had a few limitations. First, we could collect data from only five hospitals in Karachi and not all. However, these five hospitals covered all major trauma centers of the city and the only ones to have a 24/7 medico-legal officer in the hospital. Second, the data was collected from one point in time where the city was facing an unstable condition and there was a higher frequency of crimes. Since after that time, there have been security measures and violence on the streets is considerably reduced. However, in Karachi, Pakistan, this situation is highly unpredictable and it would be interesting to see the causes of traumatic arrests in the absence of crimes. Third, we wanted to analyze the predictors of survival but we could not do that since there was not a single survivor at the end of ED stay. Lastly, because of the lack of trained medical staff in the ambulances, most arrests were assessed by lay bystanders and despite using the operational definition, some of the arrests might have been misdiagnosed. Most arrests were confirmed by a physician only on arrival to the ED, and therefore, the time of arrest might be different than stated by the witness.

## Conclusion

The survival rate post out-of-hospital traumatic cardiac arrest is zero in Karachi, Pakistan. While prevention of GSW and road traffic injuries are most effective in saving lives from traumatic cardiac arrest, our data shows poor to no care prior to arrival to the hospital. There is a strong need to train the general public to deal with emergencies like these and provide timely bystander CPR, besides working to strengthen our pre-hospital care systems.

## Abbreviations

ANOVA: Analysis of variance
CPR: Cardiopulmonary resuscitation
ED: Emergency department
EMS: Emergency medical services
IQR: Interquartile range
OHCA: Out-of-hospital cardiac arrest
RTA: Road traffic accident
SD: Standard deviation
SPSS: Statistical Package for Social Scientists
